# Supplementary material for: Analysis of chi angle distributions in free amino acids via multiplet fitting of proton scalar couplings
Source: Magn Reson (Gott). 2024 Aug 19;5(2):103–20. doi: 10.5194/mr-5-103-2024 (PMC11570886; doi:10.5194/mr-5-103-2024)
Supplement: The supplement related to this article is available online at: https://doi.org/10.5194/mr-5-103-2024-supplement. [file mr-5-103-2024-supplement.zip › Syed2024/data/fit1d_fitnmr_output.tar.gz!/fit1d/gln/400/bmse000038_simulation_1_nmredata/1H/resonances_1d.pdf]

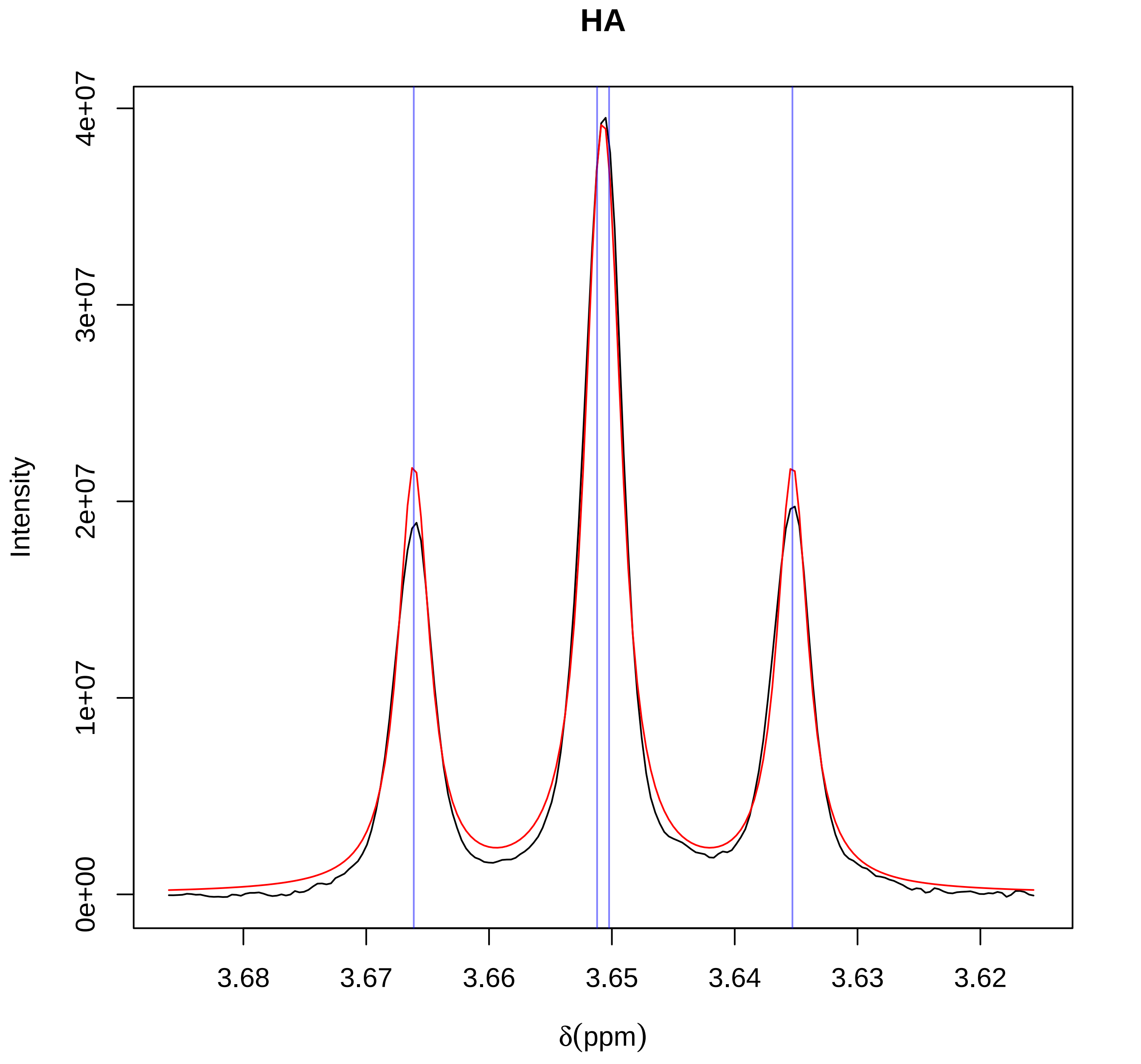

HA
Intensity
0e+00
1e+07
2e+07
3e+07
4e+07
3.68
3.67
3.66
3.65
3.64
3.63
3.62
δ(ppm)

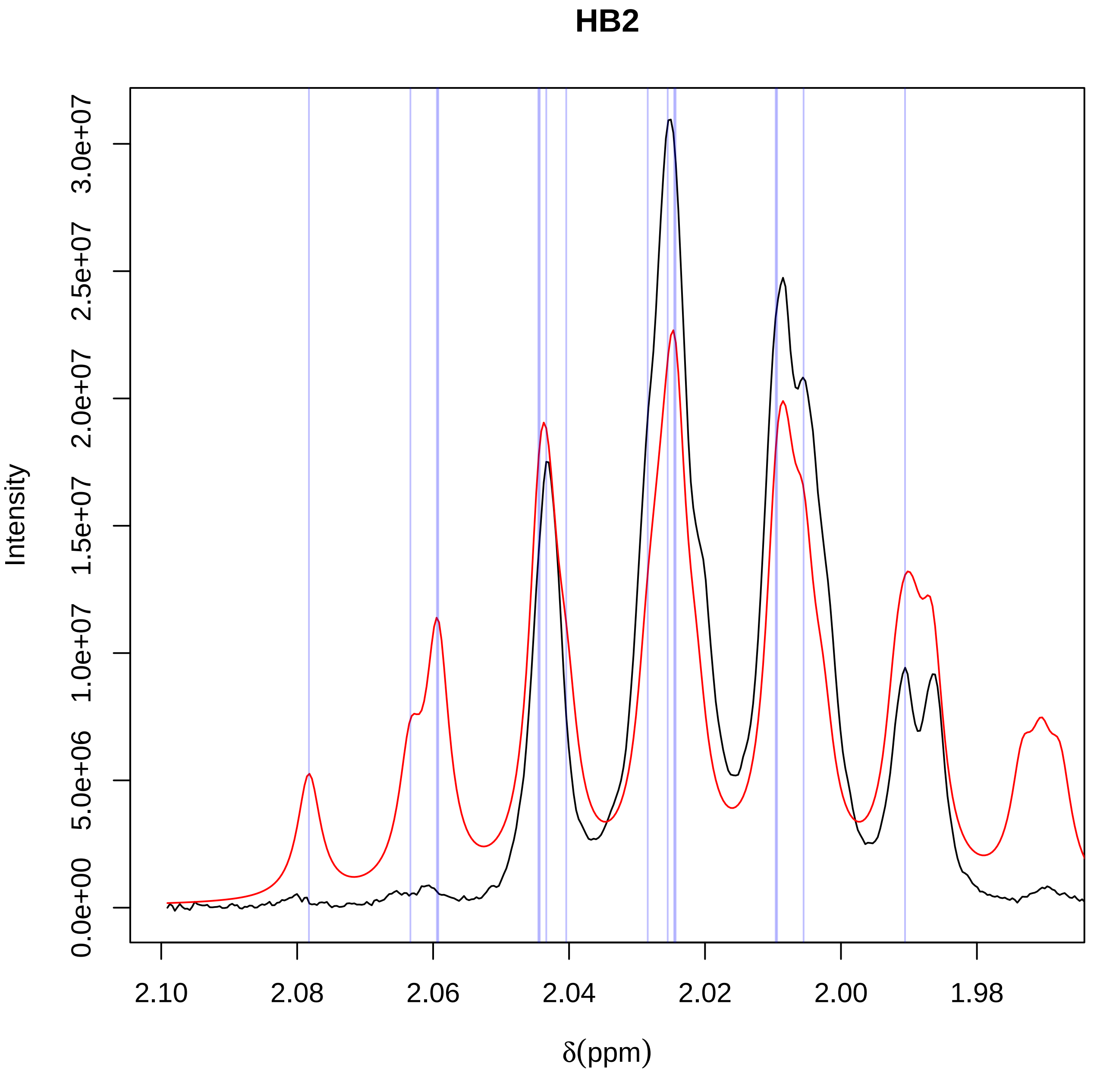

HB2
Intensity
δ(ppm)
0.0e+00
5.0e+06
1.0e+07
1.5e+07
2.0e+07
2.5e+07
3.0e+07
2.10
2.08
2.06
2.04
2.02
2.00
1.98

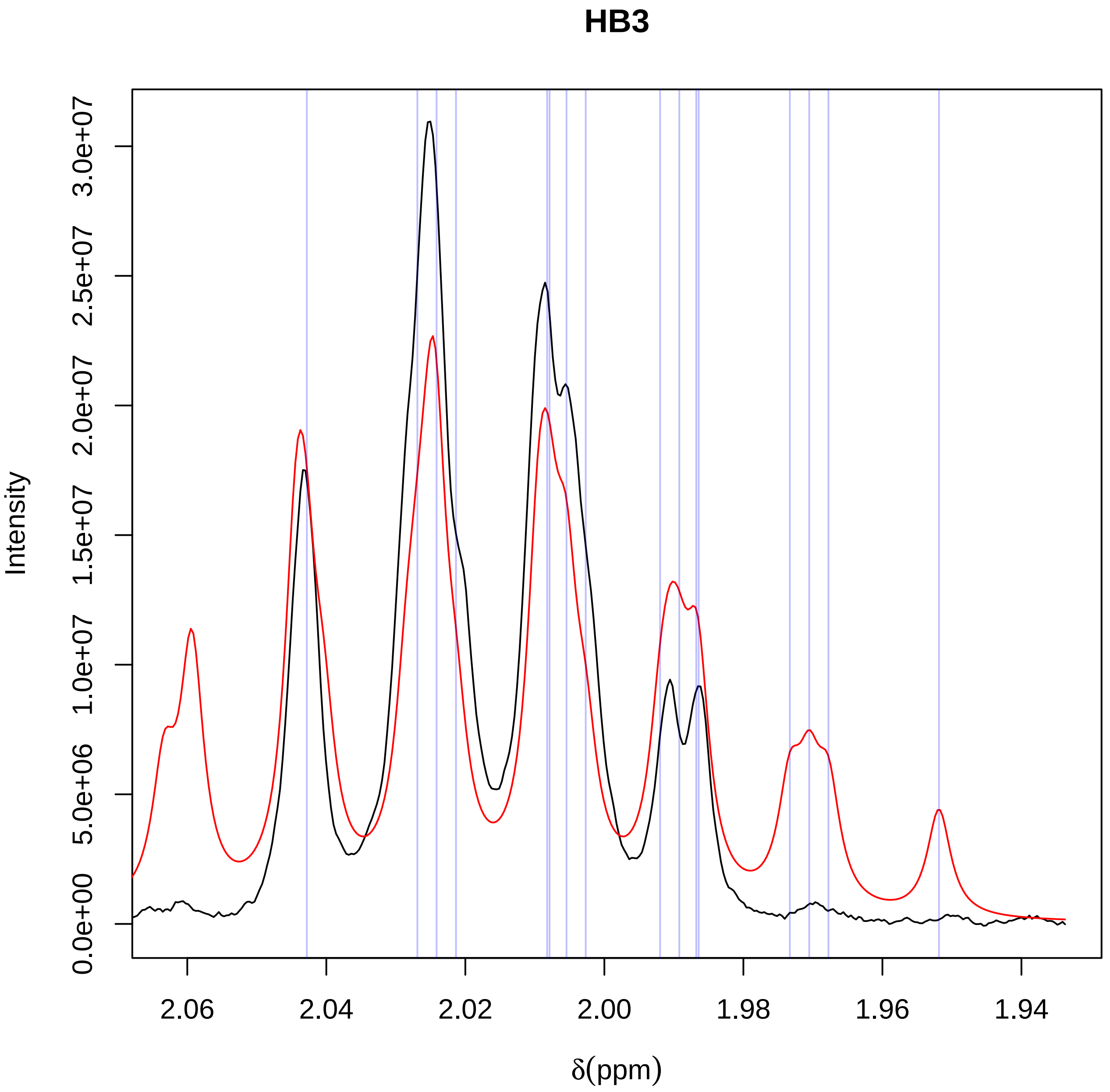
HB3
Intensity
0.0e+00
5.0e+06
1.0e+07
1.5e+07
2.0e+07
2.5e+07
3.0e+07
2.06
2.04
2.02
2.00
1.98
1.96
1.94
δ(ppm)

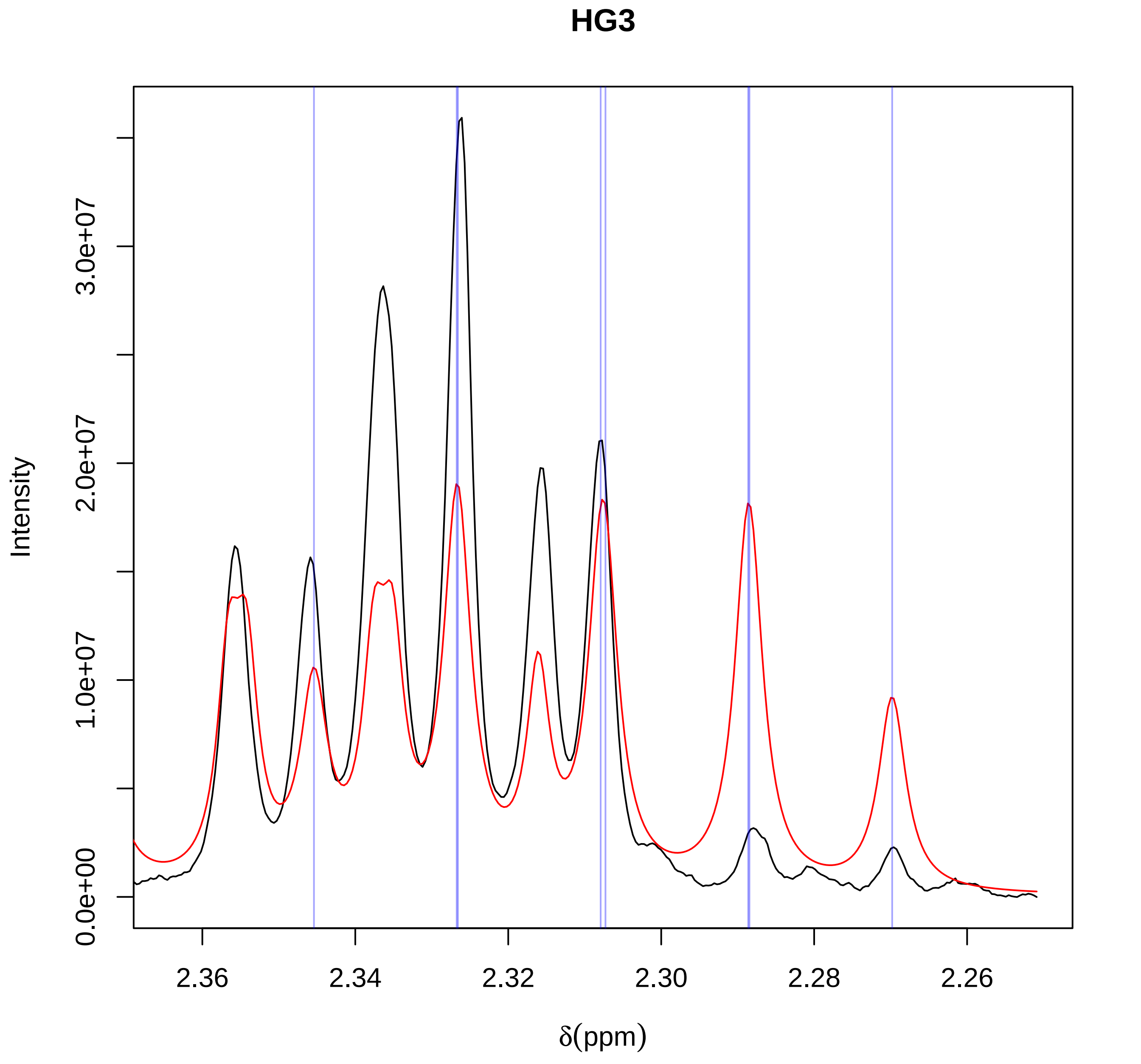
HG3
Intensity
0.0e+00
1.0e+07
2.0e+07
3.0e+07
2.36
2.34
2.32
2.30
2.28
2.26
δ(ppm)

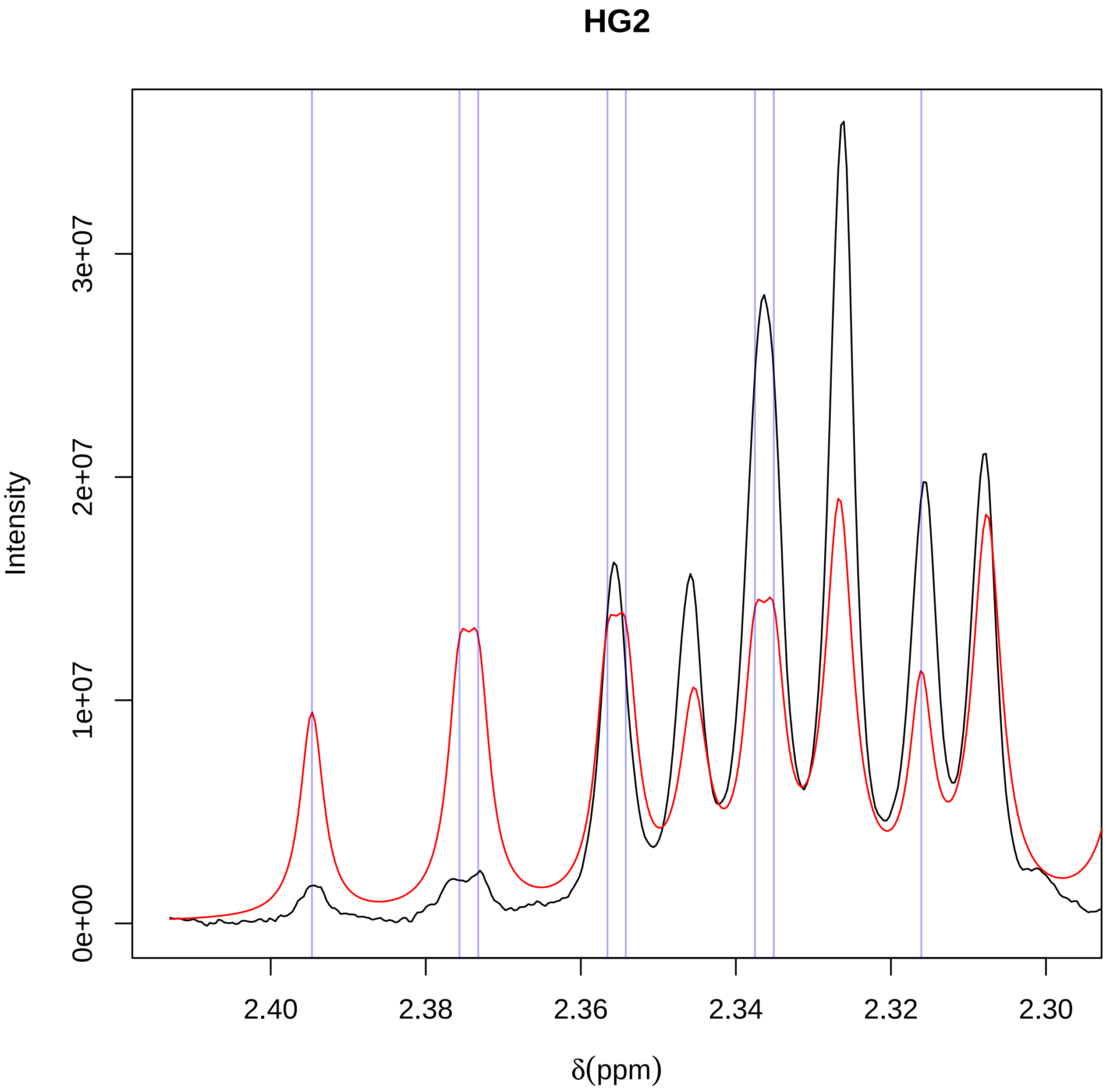
HG2
Intensity
0e+00
1e+07
2e+07
3e+07
2.40
2.38
2.36
2.34
2.32
2.30
δ(ppm)
